# Supplementary material for: Linking the gut and liver: crosstalk between regulatory T cells and mucosa-associated invariant T cells
Source: Hepatol Int. 2018 Jul 19;12(4):305–14. doi: 10.1007/s12072-018-9882-x (PMC6097019; doi:10.1007/s12072-018-9882-x)
Supplement: Supplementary file 1 — Supplementary material 1 (DOCX 3386 kb) [file 12072_2018_9882_MOESM1_ESM.docx]

**Linking the Gut and Liver: Crosstalk between regulatory T-cells and mucosa-associated invariant T-cells**

***Muhammad Atif^1,2,3^, *Suz Warner^1^, Ye H. Oo^1,3^**

^1^Centre for Liver Research and National Institute of Health Research Liver Biomedical Research Centre Birmingham, Institute of Immunology and Immunotherapy, University of Birmingham, UK; ^2^Academic Department of Surgery, University of Birmingham, UK; ^3^Liver Transplant and Hepatobiliary Unit, University Hospital of Birmingham NHS Foundation Trust, UK

*Both authors contributed equally

**Contact Information:**

Muhammad Atif: [mxa635@student.bham.ac.uk](mailto:mxa635@student.bham.ac.uk) Tel: +44 (0) 121 414 2246

Suz Warner: [suz.warner@bham.ac.uk](mailto:suz.warner@bham.ac.uk) Tel: +44 (0) 121 414 2246

Ye Oo: [y.h.oo@bham.ac.uk](mailto:y.h.oo@bham.ac.uk) Tel: +44 (0) 121 414 2246 **(Corresponding Author)**

Figures: 3

Words: 4470 (excluding Abstract and References)

**Funding Information**

Mr Muhammad Atif is the recipient of the National Institutes for Health Research (NIHR) Academic Clinical Fellowship in General Surgery. Dr Ye Oo is supported by the Medical Research Council, Queen Elizabeth Hospital Charity, National Institute of Health Research Birmingham Biomedical Research Centre, and Medical Research Council Clinician Scientist Award (Grant number—G1002552).

**Abstract**

The gut-liver axis is increasingly considered to play a vital part in the progression of chronic inflammatory gut and liver diseases. Hence, a detailed understanding of the local and systemic regulatory mechanisms is crucial to developing novel therapeutic approaches. In this review, we discuss in-depth the roles of regulatory T-cells (Tregs) and mucosal-associated invariant T-cells (MAITs) within the context of inflammatory bowel disease, primary sclerosing cholangitis, and non-alcoholic steatohepatitis. Tregs are crucial in maintaining peripheral tolerance and prevent autoimmunity. MAIT cells have a unique ability to rapidly recognize microbial metabolites and mount a local immune response and act as a ‘biliary firewall’ at the gut and biliary epithelial barrier. We also outline how current knowledge can be exploited to develop novel therapies in order to control the propagation of chronic gut- and liver-related inflammatory and autoimmune conditions. We specifically focus on the nature of the Tregs cell therapy product and outline an adjunctive role for low-dose IL-2. All in all, it is clear that translational immunology is at a crucial crossroads. The success of ongoing clinical trials in cellular therapies for inflammatory gut and liver conditions could revolutionize the treatment of these conditions and the lives of our patients in the coming years.

**Introduction**

The gut-liver axis is considered to play a key role in immune-mediated diseases such as autoimmune sclerosing cholangitis/primary sclerosing cholangitis (AISC/PSC) and inflammatory bowel disease (IBD). The underlying causes of these diseases and their progression are multifactorial as the gut comprises of a unique microbiome, ingested nutrients, the mucosal immune system as well as the gut-portal vein barrier. [1-4] As there is yet no effective therapeutic for both IBD and AISC, patients can develop intestinal failure, colonic malignancy and in cases of AISC, the disease progress to fibrosis, cirrhosis and hepatocellular carcinoma (HCC) and cholangiocarcinoma.[3-5] Hence, it is more important than ever to understand the gut-liver axis and in particular, targeted anti-inflammatory pathways.

The role of regulatory T-cells (Tregs) has been extensively explored in autoimmune-mediated inflammatory gut and liver diseases such as IBD and AISC/PSC albeit mainly within pre-clinical settings.[6-10] Tregs are crucial in preventing autoimmunity as they have an anti-inflammatory property that can suppress effector T-cell subsets. [11] Additionally, alongside Tregs, there are other immune cells in the gut and liver microenvironments such as myeloid-derived suppressor cells and tolerogenic dendritic cells that could also provide a similarly beneficial anti-inflammatory function.

Mucosal-associated invariant T-cells (MAIT cells), which are a mucosa T-lymphocyte subset in humans. [12-14] These cells are characterized by the expression of a conserved Vα7.2 chain and CD161 receptor expression but are restricted by the major histocompatibility complex class 1-related molecule (MR1). [13, 15] This is important as it infers that MAIT cells are an evolutionarily evolved T-cell subset that has the capacity to directly recognize a narrow repertoire of bacterial-derived vitamin B metabolites.[12] Additionally, their location in the gut mucosa and continuity with the biliary epithelium is especially pertinent as these are the two sites of bacterial entry into the gut and liver microenvironments. [13]

**Gut-Liver Axis**

Due to its unique embryonic development, the human liver, which originates from the endoderm is connected to the gut via both the biliary tracts and portal vein. As a result of the multiple localized components of the gut (eg. microbiome, nutrients, metabolites, mucosal immunity, gut-portal vein barrier), it has not always been possible to identify and dissect a specific cause of immune-mediated chronic inflammatory gut and liver disease processes.[1, 2] More importantly, as the liver is the first recipient of these gut-sourced components, any homeostatic alteration in the profile of these gut components can directly impact the liver’s own homeostatic physiological processes. [1, 3] When one considers the multiple functions of the liver (eg. glucose homeostasis, lipid transport, protein synthesis as well as immune surveillance), it is comprehendible how the gut can impact both hepatic and systemic physiology.

The gut microbiome has more than a trillion microbes that chiefly comprise of 3 bacterial phyla; *Bacteroidetes*, *Actinobacteria*, and *Firmicutes*. [1, 3] Whilst these phyla exist in individual-specific proportions in healthy individuals, their altered proportions have been reported in a number of patients with chronic inflammatory gut and liver diseases.[1, 3, 16] For example, in patients with non-alcoholic steatohepatitis (NASH), a condition closely associated with obesity, an inverted Bacteroidetes/Firmicutes ratio has been reported compared to those obese patients without NASH. [5, 17] Importantly, whether this dysbiosis is a cause or effect of inflammatory gut/liver disease is yet unknown. These microbes are responsible for digesting dietary polysaccharides to form monosaccharides, free fatty acids (FFAs) such as acetate, propionate, and butyrate as well as reactive oxygen species (ROS) and ethanol as by-products. [1, 17] The parallel effects of ROS and ethanol are to both interfere with mucosal parenchymal and immune cell function through oxidative stress and damage the tight junctions that enclose the intestinal epithelial layer, which increases intestinal permeability. This increased intestinal permeability allows for further diffusion of danger-associated molecular patterns (DAMPs), FFAs, ROS across the epithelial layer and into the portal venous supply. [3, 18] The intraportal translocation of these molecules to the liver and subsequent transmigration along the sinusoids provides chronic inflammatory stimulus. It is this induced inflammatory process that interferes with the liver’s parenchymal and immune cell functions and thereby, propagates chronic liver disease.

Conceptually, it is vital to keep in mind that although the liver is the destination of gut-sourced compounds, the liver itself can also influence gut absorptive function through both primary and secondary bile acids, bile salts and immunoglobulins secretion. [19, 20] Bile acids act via the farsenoid X receptor (FXR) to alter gene expression of intracellular programs responsible for inflammation as well as metabolism of bile acids, glucose, cholesterol, and lipids. [20] This has implications for gut microbes as it alters substrate availability for metabolism and thereby alters their own ability to metabolize polysaccharides as described above. Additionally, liver-secreted immunoglobulins (eg. IgA) are responsible for forming a protective biofilm that reinforces the intestinal epithelial layer.[21] Hence, any hepatic pathology can lead to alterations in the secretion of biliary acids and immunoglobulins and consequently, impact gut function.

**Regulatory T cells**

Thymic Tregs are a subset of CD4 T cells that are characterized by high-level expression of interleukin-2 receptor alpha chain (CD25) and low expression of interleukin 7 receptor alpha chain (CD127). [22, 23] Thus, CD4+CD25+CD127low is the typical phenotype of thymic Tregs. Treg also express the Forkhead Box P3 (Foxp3) transcription factor, which is a master regulator of Treg development and function. [24, 25] Indeed, such is its importance that loss of Foxp3 is associated with the development of the autoimmune syndrome; IPEX (immune dysfunction, polyendocrinopathy, enteropathy, X-linked) in humans and the *scurfy* phenotype in mice. [26, 27]

Tregs exist as 2 main subsets within the peripheral microenvironment; thymus-derived (tTregs) and peripherally-induced Tregs (pTregs). [28, 29] The tTregs mature in the thymus and exit into the peripheral circulation as *bona fide* Tregs (Foxp3+) however, the pTregs are derived from differentiated CD4+CD25+Foxp3- T-cells upon T-cell receptor (TCR) stimulation in the presence of transforming growth factor beta (TGF-β) and interleukin-2 (IL-2). [28, 29] Both subsets have been identified in the gut and liver and both exhibit immunosuppressive and homeostatic functions. [8, 30, 31] However, in order to exhibit these functions in their respective microenvironments, Tregs express chemokine receptors, which facilitate their migration and homing to the specific and relevant tissue directed by the corresponding chemokines which act as postcodes for the receptors (eg. CCR9/α4β7 and CCL25/MadCAM for gut homing, CXCR3 and CXCL9-11 for inflammatory liver diseases and specifically, CCR9, αEβ7, CCL25/E cadherin in AISC and PSC. [6, 8, 9, 32] Indeed, we have shown that inflamed liver sinusoids have increased expression of CXCR3 ligands such as CXCL9 and CXCL10 which further facilitates recruitment of CXCR3 expressing Tregs in order to control the hepatic inflammatory response. [6] Tregs remain as the main regulatory immune cells to maintain hepatic tolerance, which they achieve through a range of functional markers such as CTLA-4, CD39, LAG-3 and secretion of IL-10 amongst others.[33, 34] **(Figure 1)**

**
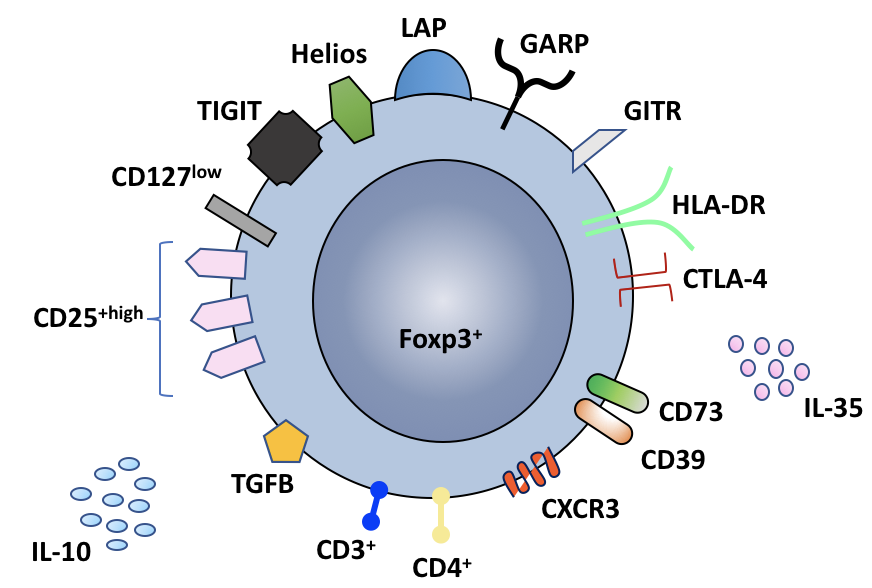
**

**Figure 1**: Tregs are recognised as CD4+CD25+CD127lo (extracellularly) and Foxp3+ (intracellularly). They express a range of other phenotypic markers such as TIGIT (T cell immunoreceptor with Ig and ITIM domains), Helios, LAP (Latency-associated peptide), GARP (Glycoprotein A repetitions predominant), GITR (Glucocorticoid-induced tumour necrosis factor receptor-related protein), HLA-DR, CTLA-4 (cytotoxic T-lymphocyte-associated protein 4), CD73, CD39 and CXCR3. Upon activation, Tregs release interleukin-10 (IL-10) and interleukin 35 (IL-35).

Although there have been extensive pre-clinical studies, the *in vivo* Treg mechanisms of action remain undefined. The current consensus is that Tregs utilize multiple mechanisms in order to perform their functions. [35, 36] For a detailed overview of these mechanisms, we refer you to excellent reviews by Shimon Sakaguchi and Makoto Miyara. [35, 36]

**MAIT Cells**

MAIT cells are found in abundance within peripheral tissues such as the gut and liver, however, their exact roles in the pathogenesis of autoimmune-related diseases in these compartments have not been fully elucidated. [15, 37, 38] MAIT cells are defined as CD3+, Vα 7.2 +, CD161+ T lymphocytes **(Figure 2)**. [39, 40] Human MAIT cells are predominantly of the CD8+ effector memory phenotype. Although double negative MAIT cells (CD4- CD8-) are present in modest numbers and the CD4+ T lymphocytes make up only a minority of this subset in the peripheral circulation, the relative proportions of MAIT cells alter in chronic liver disease. [15] [39, 41] This diversity leads further credence to their differing roles in the microenvironment depending on their phenotype.

**
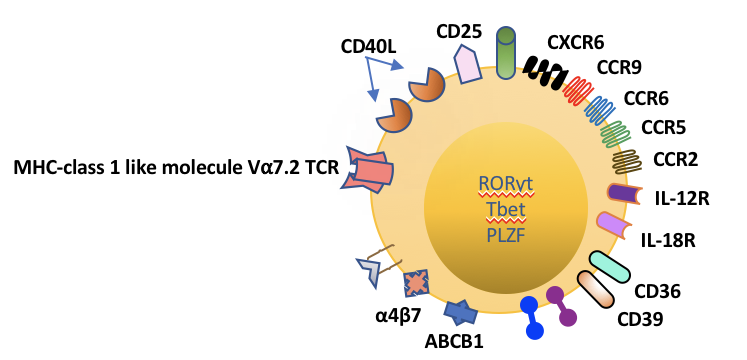
Figure 2:** Intrahepatic MAIT cells are characterized by expression of CD3+, TCR Vα7.2, and CD161. Their characteristic intracellular markers are Tbet, RORγT, and PLZF. They also express a range of receptors and markers as visualized in this figure. Upon activation, MAIT cells release the cytokines TNFα, IFNγ, and IL-17. They can also release granzymes and perforin.

Bacteria, yeast and viruses can all stimulate a MAIT-cell mediated effector response through their DAMPs.[42-44] This role of DAMPs in patients with inflammatory gut and liver disease is evidenced by their increased levels both in the inflammatory microenvironment and peripheral blood.[45-47]. In particular, Kjer-Nielson et al showed that products of bacteria-derived vitamin B2 metabolism (riboflavin metabolites) could be presented to Vα7.2 expressing MAIT cells in an MR1-dependent manner by antigen-presenting cells (APCs) such as monocytes, dendritic cells, B-cells.[12] These APCs could have been infected by variety of bacteria and fungi, including Mycobacterium tuberculosis, Salmonella typhimurium, Escherichia coli, Staphylococcus aureus and Candida albicans.[43] This presentation would lead to activation of MAIT cells and trigger a prompt inflammatory response by cytokines, granzymes secretion, and degranulation; thereby eradicating the early localized infection. [48]. Interestingly, MAIT cells do not recognise all bacteria, prime examples being Enterococcus faecalis, Listeria monocytogenes and group A Streptococcus, implicating these strains lack the ability to produce the relevant DAMPs.[43]

MAIT cells can also be activated in an MR1-independent manner by viruses (eg. hepatitis C (HCV), influenza and dengue) through the interleukin receptors IL12R and IL18R, which are present on their cell surface [44]. Their effector response was evidenced by upregulation of granzyme B expression. Interestingly, patients with hepatitis C receiving Pegylated interferon (IFN-α) as part of their treatment regime demonstrated increased MAIT cell expression of the activation marker CD69. which was associated with a higher sustained virologic response (SVR) as well. This indicates that MAIT cell activity may enhance the host immune response to this virus.

Intracellularly, MAIT cells express several transcription factors, most notably retinoic acid-related orphan receptor *gamma t* (RORƴt), which controls IL-17 production and T-bet , which controls TNFα and IFNγ secretion. [49] MAIT cells also possess a set of homing chemokine receptors (CCR2, CCR5, CCR6, CCR9, and CXCR6), which enables their recruitment or ‘trafficking’ to specific sites of inflammation.[49] Upon activation, MAIT cells produce TNFα, IFNγ, IL-2, IL-17 and release cytotoxic granzymes and perforin; which rapidly induce cytolysis and death of target cells. [49]

**Inflammatory Bowel Disease (IBD)**

The role of Tregs has been extensively studied in IBD. [8, 9] This is an autoimmune condition predominantly of the gut characterized by dysregulation of the mucosal immune system.[9, 50, 51] This condition is pertinent to the gut-liver axis as approximately 66-75% of PSC patients will also develop IBD.[40, 52] Whilst the underlying reasons for this cross-over are unknown, it potentially indicates molecular mimicry between gut-specific and biliary-specific immune cells. Additionally, there may be a role for bi-directional trafficking of pro-inflammatory immune cells between the gut and liver. Indeed, if true, this would complement novel findings from our Department describing the role of gut-homing memory mucosal lymphocytes albeit within the context of PSC. [53-55] Hence, any therapeutic in IBD/PSC must traffic to 2 different tissue sites in order to achieve disease control.

From an IBD pathogenesis perspective, gut barrier dysfunction in IBD facilitates increased exposure of bacterial products to local and lymphatic APCs, which propagates a local inflammatory response consisting of effector T-cells (Th1, Th2, Th17) and Tregs. [56, 57] The role of Tregs is pertinent as although they are found within the gut mucosa of healthy patients, they exist in higher levels in the inflamed tissue of patients with IBD. [58] Additionally, studies have reported these Tregs to be less functional than those of healthy patients, which has implications for their ability to control the local inflammatory response. [9, 58]

Tregs in IBD have been studied through multiple murine models of colitis such as chemically-induced (eg. dextran-sulfate sodium, TNBS) and transgenic (eg. IL-2, STAT3 KO). [59, 60] These models have been critical in demonstrating that adoptive transfer of Tregs can abrogate colonic inflammation. [60] Depending on the model used, the underlying mechanisms have been purported to involve Treg contact with pro-inflammatory APCs in the gut lymph nodes and IL-10 secretion. [8, 51, 59]

Hence, this role of Tregs as having anti-inflammatory potential in IBD could be harnessed in the form of a cellular therapy to treat IBD patients with disease refractory to current medical regimens. [9, 61] Additionally, as there is yet no specific antigen which is known to initiate or propagate the pathogenesis of IBD, the Treg cellular therapy product best be better suited to an autologous polyclonal version. However, one must be mindful as to the relative efficacy of polyclonal Tregs as opposed to gut ‘antigen-experienced’ Tregs.[62] This has been taken further by Lord, Lombardi, and colleagues who identified the naïve Tregs (CD4+CD25+CD127loCD45RA+) population as the ‘most appropriate population’ to use in a trial for IBD as they remain lineage stable following expansion. [61] This was on the basis of stable expression of the Foxp3 gene (key for Treg function and development), CCR7 and α4β7 (gut homing markers). They are presently undertaking the TRIBUTE trial to investigate Treg therapy in Crohns' disease (NCT03185000**)**. [61]

Interestingly, in IBD, MAITs are found to be decreased in number in the blood but increased at sites of inflammation such as the ileum in Crohns’ - thus supporting the concept of cell trafficking. MAITs are, however, also found in the lamina propria of healthy individuals. [41, 63] Some investigators observed that MAITs in IBD patients were expressing higher levels of caspase, which indicates their apoptotic nature within this disease. [12] [64] Whether this results from cellular exhaustion following activation and is an anergic phenotype or reflects cellular dysfunction is presently under investigation by our group.

Additionally, cytokines, chemokines, and metabolites within the gut microenvironment can further facilitate favorable conditions for activation of MAITs. This is important as the intestinal mucosa of patients with Crohn’s is abundant in IL-12 and IL-18, which enables MAIT cell activation in an MR1-independent manner. [41] Upon activation, MAITs are capable of releasing inflammatory cytokines such as IFNγ. However, patients with Crohn’s have an altered cytokine production upon activation; involving decreased IFNγ and increased IL-17. [65] Whether these findings are incidental descriptive observations or demonstrate a direct role for MAITs in Crohn's is yet unknown. Intriguingly, recent reports comment on a protective role of IL-17 in the intestinal mucosa. [66] This was clearly demonstrated in a clinical trial of secukinumab, an anti-IL17 antibody whose administration resulted in exacerbation and flare-up of Crohn's in patients. [63, 65] Put together, perhaps this altered cytokine production in IBD reflects the fact that during chronic inflammation the production of IL-17 by MAIT cells together with other IL-17 producing immune cells provides attenuation of the inflammatory activity operating therefore as part of the body's immune resolution mechanism to control the disease.

In general, patients with IBD have an altered gut microbiome.[67] This is potentially pertinent to MAITs are they are activated by bacteria and fungi that produce ligands of the riboflavin synthetic pathway. MAITs can recognize ligands presented by APCs infected by a variety of microbes, including M. tuberculosis, S. typhimurium, E. coli, S. aureus and C. albicans. Importantly, not all bacteria have the capacity to activate MAIT cells: Enterococcus faecalis, Listeria monocytogenes and group A Streptococcus do not activate MAIT cells, suggesting these strains lack the offending antigen. [15, 49, 68] Hence, we would hypothesize the gut dysbiosis can directly alter MAIT cell function. [37] A recent report by Klenerman and colleagues also suggested the role of MAIT cells in HCV, dengue and influenza infection thus, demonstrating that bacterial/viral infection could lead to activation of MAITs both in the gut and liver. [44] This line of inquiry is presently under investigation by our group to dissect the gut-liver axis.

**Biliary and Gut Epithelial Immunity**

The portal vein is the main mode of transport of gut-derived antigens, endotoxins and nutrients to the liver. [69] Indeed, the gut and liver need to be able to differentiate continuously between self- and non-self-antigens to maintain tolerance. At the same time, both organs have to rapidly respond to an array of potentially pathogenic microbes to mount the immune response. The direct link between the gut and liver is attributed to have a potential causal role into why IBD patients commonly also have PSC. [40, 52] Although there are multiple animal models to study each condition in isolation, the respective animal models to accurately reflect dual pathologies concomitantly are still under development. [70]

As the liver is the first destination of all gut-derived compounds, the liver’s Kupffer cells have been referred to as a vascular ‘firewall’ that protects the host against gut and biliary epithelial DAMP exposure. [49] Our group has demonstrated that MAIT cells are found predominantly around the intrahepatic bile ducts in the vicinity of the portal tracts. [71] This finding was seen both in healthy ‘donor’ livers and explanted livers from patients with inflammatory liver diseases. Additionally, the MAIT cells from the latter group of patients actively secreted TNFα, IL-17 and produced granzyme B when bacteria were presented via MR1 to Vα 7.2 expressing intrahepatic antigen-presenting B-cells. **(Figure 3)** We thus proposed that intrahepatic MAIT cells are ideally located to act not only as part of the firewall conferring protection around in the portal tract as a ‘biliary firewall’ by protecting ascending infection via biliary tree. [13] This immunosurveillance function in the liver is an orchestrated effort not only by intrahepatic MAIT cells, intrahepatic Tregs and Kupffer cells, dendritic cells but also by the vast array of fast-acting immune surveillance cells such as the natural killer cells, innate lymphoid cells, and gamma delta T-cells. [72, 73] Additionally, we demonstrated that biliary epithelial cells (BEC) are able to act as ‘‘non-professional” APCs when exposed to E. coli and subsequently activate MAIT cells in an MR1-dependent manner and secrete cytokines and perform degranulation. [71] **(Figure 3)** These crucial findings provide further evidence that MAIT cells have the capacity to safeguard the biliary mucosa, which is in direct continuity with the gastrointestinal tract and constant exposure to its microbiome.

**
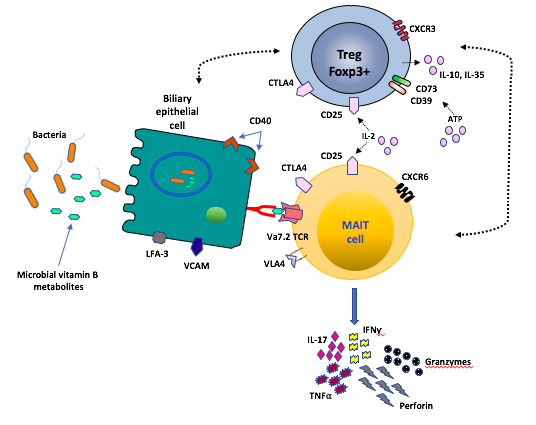
**

**Figure 3:** Demonstrating the MR1-restricted presentation to MAITs by biliary epithelial cells (BECs) and how Tregs could potentially interfere with this process. The BECs act as a ‘non-professional’ APCs as they possess the MHC Class 1-like molecule, MR1. MR1 restricts the Vα7.2 T cell receptor on intrahepatic MAIT cells to microbial-derived vitamin B metabolites. Upon activation, MAIT cells release the cytokines TNFα, INFγ, IL-17 and the degranulating proteins; granzymes and Perforin. We hypothesize that Tregs can interfere with either MAITs or BECs or both with resulting effects on the activation status of MAITs. This will have implications for the roles of Tregs as anti-inflammatory cells and MAITs as anti-microbial responders. The exact mechanisms of this interaction are currently under investigation by our group.

**NASH**

NASH is a chronic inflammatory liver condition that can progress to fibrosis, cirrhosis and even HCC. [74, 75] This condition is pertinent to the gut-liver axis as its development is intrinsically linked to a high-fat diet and dysbiosis of the gut microbiome. [1, 3] The high level of fat in the Western diet leads to hepatic steatosis and thereby changes the make-up of nutritional substrates available for local cellular metabolism. [76-78] This has direct consequences for Treg function as Tregs intrinsically rely on fatty acid oxidation as their main pathway for adenosine triphosphate (ATP) production. [79]

Moreover, the severity of NASH directly correlates with an inverted Th17/Treg ratio. [77, 80] This is augmented by a pro-inflammatory cytokine profile consisting of increased local levels of IL-6, IL-17, 21-23. [80] Studies in NASH patients who undergo extensive weight loss have demonstrated reduced levels of Th17 cells, increased Tregs and IL-10 levels. [80, 81] Whether these findings are coincidental or directly indicate a role for Th17 cells and Tregs in NASH is unknown. Due to the recent surge in metabolic surgery for NASH patients, we anticipate further evidence in future for the immunological mechanisms driving improvements in fatty liver disease and other components of the metabolic syndrome.

From the perspective of the gut microbiome, patients who develop NASH exhibit an inverted Bacteroidetes/Firmicutes phyla ratio compared to those only on a high-fat diet. [1, 3] This is important as the NASH patients exhibit a ‘leaky gut' which leads to exposure of an altered DAMPs profile to the gut mucosal immune system as well as the hepatic immune system through intraportal uptake. [45] These DAMPs can directly impact on Tregs through the TLR receptors. [82] This is supported by novel data from mouse models demonstrating a pivotal mechanism between TLR signaling, Foxp3 expression and subsequent Treg metabolism and function.[83] Stimulation of TLR1/2 increases proliferation of Tregs through glycolysis but reduces their suppressive capacity. However, Foxp3 expression does not promote glycolysis and increases the Treg suppressive capacity instead. The detailed roles of DAMPs on Tregs metabolism and function remains an ongoing line of inquiry in our group.

**Clinical Translation**

Tregs and MAITs have very distinct functions within the gut and liver and these functions could be synergistically exploited in order to achieve inflammatory disease control either in the gut mucosa or intrahepatic environment or around the peri-biliary region. **(Figure 3)** Indeed, Tregs on their own are already undergoing evaluation as a therapeutic cellular option for a range of inflammatory and autoimmune conditions within the context of early phase clinical trials. [30, 84-87] Our group too has recently completed its own Phase 1 safety trial involving polyclonal Tregs in autoimmune hepatitis. *(manuscript in press)* However, from the perspective of chronic hepatic viral infections, it is anticipated that inhibition of Treg function could be a potential therapeutic approach instead.[88, 89] Patients with chronic hepatitis B (HBV) and HCV have higher levels of Tregs in comparison to controls with treated disease. [88, 89] These Tregs have been found to inhibit CD4+ and CD8+ effector T-cell responses thus limiting *in vivo* infection resolution*.* However, successful translation of this approach in the hepatic viral setting has been limited by suboptimal understanding of the complex mechanisms underlying interactions between Tregs, effector T-cells and APCs in the hepatic viral setting.[88]

Nevertheless, in future, we anticipate greater clarity on the optimal dosing, safety and efficacy profile of Tregs in humans. As is being explored for other conditions, the nature of Tregs could be polyclonal, antigen-specific, genetically-modified (chimeric-antigen receptor; CAR, T cell receptor gene transfer), Foxp3- (Tr1 cells) or tissue-site specific (CCR7/α4β7 or CXCR3 Treg).[61, 86, 90, 91] It is likely that the final Treg product will differ depending on the disease pathology as certain autoimmune conditions already have identified autoantigens whereas others remain unknown (eg. autoimmune hepatitis type 1 vs type 2).[6] The advantage of a known autoantigen is that investigators could manufacture a tailored patient-specific and disease-specific product with CAR T-cells or CAR Treg therapy as they are more potent thus requiring fewer cells numbers and prevent global immune suppression. However, investigators will need to weigh this up against the costs of obtaining a sufficient cell yield, processing time and procurement costs along with the regulatory process to ascertain that they fulfil the requirements of a safe investigational medicine product (IMP).

In parallel, there will also be optimization of existing Treg isolation and expansion protocols under Good Manufacturing Practice (GMP) conditions.[61, 91-93] This is important as current protocols use different pharmacological agents to maximize Treg yield and function (eg. IL-2, rapamycin, retinoic acid, activator beads).[92-94] In particular, the inclusion of low dose IL-2 is advantageous as it is crucial for Treg survival and function. [95, 96] Clinical trials in graft versus host disease (GVHD) and vasculitis using low dose IL-2 as a stand-alone therapy for inflammatory and autoimmune conditions have already demonstrated increased Treg survival in these conditions and reduced disease activity.[95-98] Our group has recently reported that low dose IL-2 clinical grade can enhance Treg function in a CTLA-4 dependent manner. [95] Thus, the combination of Tregs and very low dose IL-2 could be a potentially valuable therapeutic option. This would also provide an optimal microenvironment to provide lineage stability of administered Tregs to prevent them converting to pro-inflammatory effector T-cells. [99] We anticipate that this will be crucial for inflammatory liver diseases particularly as work from our group has demonstrated their livers to be deficient in IL-2 and enriched with pro-inflammatory cytokines.[34]

In comparison to Tregs, the field of MAIT cell as a translational therapeutic application remains in its infancy and certainly, their detailed site-specific mechanisms of action still need to be delineated before therapeutic application. It is likely that the presence of MAITs in the gut and biliary epithelium facilitates their ability to rapidly recognize and respond to the increased exposure to bacterial-derived vitamin B metabolites, whose increased levels are a direct consequence of the ‘leaky gut’.[37] Patients with a ‘leaky gut’ have an increased DAMPs load both locally and in the peripheral blood, which facilitates increased recognition of vitamin B metabolites.[100] MAITs can mount a pro-inflammatory response against these bacterial-derived vitamin B metabolites to inhibit the direct effects of bacteria themselves on other immune and parenchymal cells. In the case of viral infections, work by van Wilgenburg et al supports a role for MAIT cells in response to HCV. [44] MAIT cells were activated upon acute HCV infection *in vitro* in a dose-dependent manner and in those patients on treatment for chronic HCV, increased MAIT cell activation was also identified upon treatment initiation. These activated cells inhibited replication of HCV in an IL-18-dependent manner alongside IL-12 and crucially in response to interferon alpha (IFN-α). Importantly, IFN-α is a key therapeutic in HCV treatment regimens and its ability to promote MAIT cell activation may be one of the mechanisms underlying its therapeutic role. Overall, the combination of anti-microbial effector function of MAIT cells and the anti-inflammatory effects of Tregs could be exploited in parallel to ameliorate the propagation of chronic inflammatory gut and liver diseases. **(Figure 3)**

**Conclusion**

The role of the gut-liver axis in propagating a number of inflammatory gut and liver pathologies has fascinated both clinicians and scientists. Due to the multifactorial nature of the contribution by immune cells, microbiome, diet, metabolites, and predisposition of genetic profile, the dissection of the pathologies are more challenging. However, the identification of crucial immunological mechanisms is a vital step in developing novel therapeutics. Tregs play a crucial role in preventing autoimmunity and regulating the local inflammatory microenvironment and MAITs have a role in anti-microbial immunity at the biliary and gut epithelial layers, which are consistently exposed to microbial stimulation thus both cell types could be exploited synergistically to regulate the gut-liver axis.

The holy grail remains the successful translation of this acquired knowledge to develop novel cellular therapy products and control the progression of inflammatory and autoimmune gut and liver diseases. Tregs on their own are already the subject of clinical trials by investigators worldwide. Indeed, there has never been a more exciting time to be involved in the development of cellular therapies for gut and liver diseases. The success of this novel cellular therapy approach could radically alter the therapeutic options available to clinicians and transform the lives of our patients.

**References**

1. de Faria Ghetti, F., et al., 2017. Eur J Nutr .Influence of gut microbiota on the development and progression of nonalcoholic steatohepatitis.

2. Machado, M.V. and H. Cortez-Pinto, 2012. Ann Hepatol. Gut microbiota and nonalcoholic fatty liver disease. 11(4): p. 440-9.

3. Acharya, C., S.E. Sahingur, and J.S. Bajaj, 2017. JCI Insight. Microbiota, cirrhosis, and the emerging oral-gut-liver axis. 2(19).

4. Burisch, J., et al., 2013. J Crohns Colitis. The burden of inflammatory bowel disease in Europe. 7(4): p. 322-37.

5. Boursier, J., et al., 2016. Hepatology. The severity of nonalcoholic fatty liver disease is associated with gut dysbiosis and shift in the metabolic function of the gut microbiota. 63(3): p. 764-75.

6. Oo, Y.H. and S. Sakaguchi, 2013. J Hepatol. Regulatory T-cell directed therapies in liver diseases. 59(5): p. 1127-34.

7. Yoshizawa, A., et al., 2005. Transplant Proc. The roles of CD25+CD4+ regulatory T cells in operational tolerance after living donor liver transplantation. 37(1): p. 37-9.

8. Bollrath, J. and F.M. Powrie, 2013. Semin Immunol Controlling the frontier: regulatory T-cells and intestinal homeostasis. 25(5): p. 352-7.

9. Lord, J.D., 2015. World J Gastroenterol. Promises and paradoxes of regulatory T cells in inflammatory bowel disease. 21(40): p. 11236-45.

10. Afzali, B., et al., 2007. Clin Exp Immunol. The role of T helper 17 (Th17) and regulatory T cells (Treg) in human organ transplantation and autoimmune disease. 148(1): p. 32-46.

11. Sakaguchi, S., et al., 1995. J Immunol. Immunologic self-tolerance maintained by activated T cells expressing IL-2 receptor alpha-chains (CD25). Breakdown of a single mechanism of self-tolerance causes various autoimmune diseases. 155(3): p. 1151-64.

12. Kjer-Nielsen, L., et al., 2012. Nature. MR1 presents microbial vitamin B metabolites to MAIT cells. 491(7426): p. 717-23.

13. Jeffery, H.C., et al., 2016. J Hepatol. Biliary epithelium and liver B cells exposed to bacteria activate intrahepatic MAIT cells through MR1. 64(5): p. 1118-1127.

14. Napier, R.J., et al., 2015. Front Immunol. The Role of Mucosal Associated Invariant T Cells in Antimicrobial Immunity. 6: p. 344.

15. Kurioka, A., et al., 2016. Clin Transl Immunology. MAIT cells: new guardians of the liver. 5(8): p. e98.

16. Torres, J., et al., 2018. United European Gastroenterol J. The gut microbiota, bile acids and their correlation in primary sclerosing cholangitis associated with inflammatory bowel disease. 6(1): p. 112-122.

17. Boursier, J. and A.M. Diehl, 2015. PLoS Pathog. Implication of gut microbiota in nonalcoholic fatty liver disease. 11(1): p. e1004559.

18. Bischoff, S.C., et al., 2014. BMC Gastroenterol. Intestinal permeability--a new target for disease prevention and therapy. 14: p. 189.

19. Hartmann, P., et al., 2017. Hepatology. Modulation of the intestinal bile acid-FXR-FGF15 axis improves alcoholic liver disease in mice.

20. Inagaki, T., et al., 2006 Proc Natl Acad Sci U S A Regulation of antibacterial defense in the small intestine by the nuclear bile acid receptor. 103(10): p. 3920-5.

21. Inamine, T. and B. Schnabl, 2017. J Gastroenterol. Immunoglobulin A and liver diseases.

22. Seddiki, N., et al., 2006. J Exp Med. Expression of interleukin (IL)-2 and IL-7 receptors discriminates between human regulatory and activated T cells. 203(7): p. 1693-700.

23. Liu, W., et al., 2006. J Exp Med. CD127 expression inversely correlates with FoxP3 and suppressive function of human CD4+ T reg cells. 203(7): p. 1701-11.

24. Afzali, B., et al., 2013. Eur J Immunol. CD161 expression characterizes a subpopulation of human regulatory T cells that produces IL-17 in a STAT3-dependent manner. 43(8): p. 2043-54.

25. Scotta, C., et al., 2016. Haematologica. Impact of immunosuppressive drugs on the therapeutic efficacy of ex vivo expanded human regulatory T cells. 101(1): p. 91-100.

26. Li, Y., et al., 2008. Transplantation. The presence of Foxp3 expressing T cells within grafts of tolerant human liver transplant recipients. 86(12): p. 1837-43.

27. Godfrey, V.L., et al., 1991. Proc Natl Acad Sci U S A Fatal lymphoreticular disease in the scurfy (sf) mouse requires T cells that mature in a sf thymic environment: potential model for thymic education. 88(13): p. 5528-32.

28. Povoleri, G.A., et al., 2013. Front Immunol. Thymic versus induced regulatory T cells - who regulates the regulators? 4: p. 169.

29. Shevach, E.M. and A.M. Thornton, 2014. Immunol Rev. tTregs, pTregs, and iTregs: similarities and differences. 259(1): p. 88-102.

30. Nafady-Hego, H., et al., 2010. Transplantation. The generation of donor-specific CD4+CD25++CD45RA+ naive regulatory T cells in operationally tolerant patients after pediatric living-donor liver transplantation. 90(12): p. 1547-55.

31. Nafady-Hego, H., et al., 2016. Transpl Immunol. Utility of CD127 combined with FOXP3 for identification of operational tolerance after liver transplantation. 36: p. 1-8.

32. Grant, A.J., et al., 2002. Lancet. Homing of mucosal lymphocytes to the liver in the pathogenesis of hepatic complications of inflammatory bowel disease. 359(9301): p. 150-7.

33. Rodriguez-Perea, A.L., et al., 2016. Clin Exp Immunol. Phenotypical characterization of regulatory T cells in humans and rodents. 185(3): p. 281-91.

34. Chen, Y.Y., et al., 2016. Hepatology. Human intrahepatic regulatory T cells are functional, require IL-2 from effector cells for survival, and are susceptible to Fas ligand-mediated apoptosis. 64(1): p. 138-50.

35. Miyara, M. and S. Sakaguchi, 2007. Trends Mol Med. Natural regulatory T cells: mechanisms of suppression. 13(3): p. 108-16.

36. Sakaguchi, S., et al., 2008. Cell. Regulatory T cells and immune tolerance. 133(5): p. 775-87.

37. Riva, A., et al., 2018. Gut. Mucosa-associated invariant T cells link intestinal immunity with antibacterial immune defects in alcoholic liver disease. 67(5):918-930

38. Tang, X.Z., et al., 2013. J Immunol. IL-7 licenses activation of human liver intrasinusoidal mucosal-associated invariant T cells. 190(7): p. 3142-52.

39. Eom, T., et al., 2018. J Microbiol. Current understanding of microbiota- and dietary-therapies for treating inflammatory bowel disease. 56(3): p. 189-198.

40. Eksteen, B., 2016. Clin Liver Dis. The Gut-Liver Axis in Primary Sclerosing Cholangitis. 20(1): p. 1-14.

41. Toubal, A. and A. Lehuen, 2016. J Hepatol. Lights on MAIT cells, a new immune player in liver diseases. 64(5): p. 1008-1010.

42. Ghazarian, L., S. Caillat-Zucman, and V. Houdouin, 2017. Front Immunol. *Mucosal-Associated Invariant T Cell Interactions with Commensal and Pathogenic Bacteria: Potential Role in Antimicrobial Immunity in the Child*. 8: p. 1837.

43. Gold, M.C. and D.M. Lewinsohn, 2011. Microbes Infect. *Mucosal associated invariant T cells and the immune response to infection.* 13(8-9): p. 742-8.

44. van Wilgenburg, B., et al., 2016. Nat Commun. MAIT cells are activated during human viral infections. 7: p. 11653.

45. Harte, A.L., et al., 2010. J Inflamm (Lond). Elevated endotoxin levels in non-alcoholic fatty liver disease. 7: p. 15.

46. Guo, S., et al., 2013. Am J Pathol. Lipopolysaccharide causes an increase in intestinal tight junction permeability in vitro and in vivo by inducing enterocyte membrane expression and localization of TLR-4 and CD14. 182(2): p. 375-87.

47. Nolan, J.P., 2010. Hepatology. The role of intestinal endotoxin in liver injury: a long and evolving history. 52(5): p. 1829-35.

48. Land, W.G., et al., 2016. Am J Transplant. Transplantation and Damage-Associated Molecular Patterns (DAMPs). 16(12): p. 3338-3361.

49. Hamoud, A.R., et al., 2018. Trends Endocrinol Metab Bilirubin in the Liver-Gut Signaling Axis. 29(3): p. 140-150.

50. Wood, N.J., 2011. Nat Rev Gastroenterol Hepatol. IBD: Stem cell therapy feasible, safe and beneficial for fistulizing Crohn's disease. 8(4): p. 181.

51. Harrison, O.J. and F.M. Powrie, 2013. Cold Spring Harb Perspect Biol. Regulatory T cells and immune tolerance in the intestine. 5(7).

52. Hirschfield, G.M., et al., 2013. Lancet. Primary sclerosing cholangitis. 382(9904): p. 1587-99.

53. Adams, D.H., B. Eksteen, and S.M. Curbishley, 2008. Gut. Immunology of the gut and liver: a love/hate relationship. 57(6): p. 838-48.

54. Eksteen, B., et al., 2009. Gastroenterology. Gut homing receptors on CD8 T cells are retinoic acid dependent and not maintained by liver dendritic or stellate cells. 137(1): p. 320-9.

55. Mora, J.R., et al., 2006. Science. Generation of gut-homing IgA-secreting B cells by intestinal dendritic cells. 314(5802): p. 1157-60.

56. Silva, F.A., et al., 2016. Gastroenterol Res Pract. The Immunological Basis of Inflammatory Bowel Disease. 2016: p. 2097274.

57. Sun, M., et al., 2015. Mucosal Immunol Regulatory immune cells in regulation of intestinal inflammatory response to microbiota. 8(5): p. 969-978.

58. Yamada, A., et al., 2016. World J Gastroenterol. Role of regulatory T cell in the pathogenesis of inflammatory bowel disease. 22(7): p. 2195-205.

59. Shale, M., C. Schiering, and F. Powrie, 2013. Immunol Rev CD4(+) T-cell subsets in intestinal inflammation. 252(1): p. 164-82.

60. Kiesler, P., I.J. Fuss, and W. Strober, 2015. Cell. Mol Gastroenterol Hepatol Experimental Models of Inflammatory Bowel Diseases. **1**(2): p. 154-170.

61. Canavan, J.B., et al., 2016. Gut. Developing in vitro expanded CD45RA+ regulatory T cells as an adoptive cell therapy for Crohn's disease. 65(4): p. 584-94.

62. Mathew, J.M., et al., 2018. Sci Rep. Generation and Characterization of Alloantigen-Specific Regulatory T Cells For Clinical Transplant Tolerance. 8(1): p. 1136.

63. Serriari, N.E., et al., 2014. Clin Exp Immunol. Innate mucosal-associated invariant T (MAIT) cells are activated in inflammatory bowel diseases. 176(2): p. 266-74.

64. Hiejima, E., et al., 2015. Inflamm Bowel Dis. Reduced Numbers and Proapoptotic Features of Mucosal-associated Invariant T Cells as a Characteristic Finding in Patients with Inflammatory Bowel Disease. 21(7): p. 1529-40.

65. Birkinshaw, R.W., et al., 2014. Curr Opin Immunol. MAITs, MR1 and vitamin B metabolites. **26**: p. 7-13.

66. Lee, Y., et al., 2012. Nat Immunol. Induction and molecular signature of pathogenic TH17 cells. 13(10): p. 991-9.

67. Quraishi, M.N., et al., 2017. Gut. The gut-adherent microbiota of PSC-IBD is distinct to that of IBD. 66(2): p. 386-388.

68. Reantragoon, R., et al., 2016. Asian Pac J Allergy Immunol. Mucosal-associated invariant T cells in clinical diseases. 34(1): p. 3-10.

69. Salmi, M., D. Adams, and S. Jalkanen, 1998. Am J Physiol. Cell adhesion and migration. IV. Lymphocyte trafficking in the intestine and liver. 274(1 Pt 1): p. G1-6.

70. Fickert, P., et al., 2014. J Hepatol. Characterization of animal models for primary sclerosing cholangitis (PSC). 60(6): p. 1290-303.

71. Treiner, E., 2015. Front Immunol. Mucosal-associated invariant T cells in inflammatory bowel diseases: bystanders, defenders, or offenders? 6: p. 27.

72. Jeffery, H.C., et al., 2017. PLoS One. Human intrahepatic ILC2 are IL-13positive amphiregulinpositive and their frequency correlates with model of end stage liver disease score. 12(12): p. e0188649.

73. Stuart Hunter, C.R.W., Martin S. Davey, Sofya A. Kasatskaya, Hannah C. Jeffery, Dmitriy M. Chudakov and Benjamin E. Willcox and Ye H. Oo, 2018. Journal of Hepatology. Human liver infiltrating γδ T cells are composed of clonally expanded circulating and tissue-resident populations. **Under revision**.

74. Yu, J., et al., 2016. Gastroenterol Res Pract. The Pathogenesis of Nonalcoholic Fatty Liver Disease: Interplay between Diet, Gut Microbiota, and Genetic Background. 2016: p. 2862173.

75. Townsend, S.A. and P.N. Newsome, 2017. Dig Dis. The Role of a Dedicated Non-Alcoholic Fatty Liver Disease Clinic in 2016. 35(4): p. 371-376.

76. Utzschneider, K.M. and S.E. Kahn, 2006. J Clin Endocrinol Metab. Review: The role of insulin resistance in nonalcoholic fatty liver disease. 91(12): p. 4753-61.

77. Magee, N., A. Zou, and Y. Zhang, 2016. Biomed Res Int. Pathogenesis of Nonalcoholic Steatohepatitis: Interactions between Liver Parenchymal and Nonparenchymal Cells. 2016: p. 5170402.

78. Samuel VT, S.G., 2018. Cell Metab. Nonalcoholic Fatty Liver Disease as a Nexus of Metabolic and Hepatic Diseases. 27(1): p. 22-41.

79. Wawman, R.E., H. Bartlett, and Y.H. Oo, 2017. Front Immunol. Regulatory T Cell Metabolism in the Hepatic Microenvironment. 8: p. 1889.

80. Rau, M., et al., 2016. J Immunol. Progression from Nonalcoholic Fatty Liver to Nonalcoholic Steatohepatitis Is Marked by a Higher Frequency of Th17 Cells in the Liver and an Increased Th17/Resting Regulatory T Cell Ratio in Peripheral Blood and in the Liver. 196(1): p. 97-105.

81. Paquissi, F.C., 2016. Front Immunol. Immune Imbalances in Non-Alcoholic Fatty Liver Disease: From General Biomarkers and Neutrophils to Interleukin-17 Axis Activation and New Therapeutic Targets. 7: p. 490.

82. Liu, G. and Y. Zhao, 2007. Immunology. Toll-like receptors and immune regulation: their direct and indirect modulation on regulatory CD4+ CD25+ T cells. 122(2): p. 149-56.

83. Gerriets, V.A., et al., 2016. Nat Immunol. Foxp3 and Toll-like receptor signaling balance Treg cell anabolic metabolism for suppression. 17(12): p. 1459-1466.

84. Heinrichs, J., et al., 2016. J Immunol. Res Ther Regulatory T-Cell Therapy for Graft-versus-host Disease. 1(1): p. 1-14.

85. Romano, M., et al., 2017. Transpl Int. Treg therapy in transplantation: a general overview. 30(8): p. 745-753.

86. Bluestone, J.A., et al., 2015. Sci Transl Med. Type 1 diabetes immunotherapy using polyclonal regulatory T cells. 7(315): p. 315ra189.

87. Marek-Trzonkowska, N., et al., 2014. Clin Immunol. Therapy of type 1 diabetes with CD4(+)CD25(high)CD127-regulatory T cells prolongs survival of pancreatic islets - results of one year follow-up. 153(1): p. 23-30.

88. Karkhah, A., M. Javanian, and S. Ebrahimpour, 2018. Infect Genet Evol. *The role of regulatory T cells in immunopathogenesis and immunotherapy of viral infections.* 59: p. 32-37.

89. Rouse, B.T., P.P. Sarangi, and S. Suvas, 2006. Immunol Rev. *Regulatory T cells in virus infections.* 212: p. 272-86.

90. Gregori, S., M. Battaglia, and M.G. Roncarolo, 2008. Novartis. Found Symp Re-establishing immune tolerance in type 1 diabetes via regulatory T cells. 292: p. 174-83; discussion 183-6, 202-3.

91. MacDonald, K.G., et al., 2016. J Clin Invest. Alloantigen-specific regulatory T cells generated with a chimeric antigen receptor. 126(4): p. 1413-24.

92. Wiesinger, M., et al., 2017. Front Immunol. Good Manufacturing Practice-Compliant Production and Lot-Release of Ex Vivo Expanded Regulatory T Cells As Basis for Treatment of Patients with Autoimmune and Inflammatory Disorders. 8: p. 1371.

93. Peters, J.H., et al., 2008. PLoS One. Clinical grade Treg: GMP isolation, improvement of purity by CD127 Depletion, Treg expansion, and Treg cryopreservation. 3(9): p. e3161.

94. Brunstein, C.G., et al., 2016. Blood. Umbilical cord blood-derived T regulatory cells to prevent GVHD: kinetics, toxicity profile, and clinical effect. 127(8): p. 1044-51.

95. Jeffery, H.C., et al., 2017. Clin Exp Immunol. Low-dose interleukin-2 promotes STAT-5 phosphorylation, Treg survival and CTLA-4-dependent function in autoimmune liver diseases. 188(3): p. 394-411.

96. Matsuoka, K., et al., 2013. Sci Transl Med. Low-dose interleukin-2 therapy restores regulatory T cell homeostasis in patients with chronic graft-versus-host disease. 5(179): p. 179ra43.

97. Koreth, J., et al., 2011. N Engl J Med. Interleukin-2 and regulatory T cells in graft-versus-host disease. 365(22): p. 2055-66.

98. Whitehouse, G., et al., 2017. Proc Natl Acad Sci U S A. IL-2 therapy restores regulatory T-cell dysfunction induced by calcineurin inhibitors. 114(27): p. 7083-7088.

99. Zhou, X., et al., 2009. Nat Immunol. Instability of the transcription factor Foxp3 leads to the generation of pathogenic memory T cells in vivo. 10(9): p. 1000-7.

100. Takiishi, T., C.I.M. Fenero, and N.O.S. Camara, 2017. Tissue Barriers. Intestinal barrier and gut microbiota: Shaping our immune responses throughout life. 5(4): p. e1373208.
